# Roadside opioid testing of drivers using oral fluid: the case of a country with a zero tolerance law, Spain

**DOI:** 10.1186/s13011-017-0108-3

**Published:** 2017-05-10

**Authors:** Inmaculada Fierro, Mónica Colás, Juan Carlos González-Luque, F. Javier Álvarez

**Affiliations:** 1 Institute for Alcohol and Drug Studies, Pharmacology and Therapeutics, Faculty of Medicine, & CEIC/CEIm Hospital Clinico Universitario de Valladolid, 47005 Valladolid, Spain; 2 Directorate General for Traffic (Dirección General de Tráfico), 28071 Madrid, Spain

**Keywords:** Drug abuse, Oral Fluid, Automobile driving, Drivers, Heroin, Methadone, Saliva, Opioid addiction, Substance abuse detection, Street drug testing

## Abstract

**Background:**

Opioids can impair psychomotor performance, and driving under the influence of opioids is associated with an increased risk of accidents. The goals of this study were i) to determine the prevalence of opioids (heroin, morphine, codeine, methadone and tramadol) in Spanish drivers and ii) to explore the presence of opioids, more specifically whether they are used alone or in combination with other drugs.

**Methods:**

The 2008/9 DRUID database regarding Spain was used, which provided information on 3302 drivers. All drivers included in the study provided a saliva sample and mass-chromatographic analyses were carried out in all cases. To determine the prevalence, the sample was weighted according to traffic intensity. In the case of opioid use combinations, the sample was not weighted. The detection limit for each substance was considered a positive result.

**Results:**

The prevalence of opioids in Spanish drivers was 1.8% (95% CI, 1.4–2.3). Polydrug detection was common (56.2%): of these, in two out of three cases, two opioids were detected and cocaine was also detected in 86% of the cases. The concentration (median [Q1-Q3] ng/ml) of the substances was low: methadone 1.71 [0.10–15.30], codeine 40.55 [2.10–120.77], 6-acetylmorphine 5.71 [1.53–84.05], and morphine 37.40 [2.84–200.00]. Morphine was always detected with 6-acetylmorphine (heroin use).

**Conclusions:**

Driving under the influence of opioids is relatively infrequent, but polydrug use is common. Our study shows that 6 out of 10 drivers with methadone in their OF (likely in methadone maintenance programs) are using other substances. This should be taken into account by health professionals in order to properly inform patients about the added risks of mixing substances when driving.

## Background

Opioids can impair driving-related psychomotor performance, and the likelihood of being seriously injured or killed in an accident while positive for opioids is within the range of medium increased risk (Relative Risk in the range of 2–10) [1, 2].

The legal framework concerning driving under the influence of drugs varies worldwide, but three approaches are used [3], page 4: i) “Zero tolerance laws make it unlawful to drive with any amount of specified drugs in the body; ii) Impairment laws make it unlawful to drive when the ability to drive has become impaired following drug use, often described as being “under the influence” or similar terms; iii) Per se laws make it unlawful to drive with amounts of specified drugs that exceed the maximum set concentration”.

Spain has a dual system: a zero tolerance law for driving with the presence of any amount of any illicit drugs (observed impairment is not necessary, and there is an administrative sanction of 1000€ along with the loss of 6 driver’s license points) and an impairment law (when impairment is observed, penal sanction) [4]. However, in other countries, such as the United Kingdom [5] or Norway [6], the per se law applies and specified limits for some prescribed medicines and illicit drugs have been established.

The biological matrix used for the analysis is an issue of relevance. Some countries use blood, and others use saliva (oral fluid (OF)). The equivalence between blood and OF concentrations is an object of concern [7, 8]. In the DRUID project, equivalent analytical cut-off values for both blood and OF were given for key substances [1, 2].

Roadside drug testing is a current practice in Spain, with OF as a matrix for screening. Current devices can detect some groups of substances, including one for opioids (generally heroin, morphine and codeine), and some devices have kits with the ability to detect methadone. The Dräger DrugTest® 5000, DrugWipe® and Alere™ DDS®2 Mobile Test System [4] are currently used in Spain. The substances (and main metabolites) that are detected by each device and the cut-offs are not necessarily the same. When a roadside screening test is positive, a second OF sample is taken and later submitted to an accredited toxicological laboratory for confirmation and quantification.

Spanish law states that when the substance detected is medically prescribed and is in accordance with the Summary of Product Characteristics, sanctions do not apply (as long as no other illicit substance is detected). This requires a physician’s report. Although the system is different in the UK (where the per se law is in place) [9], UK legislation also provides for statutory “medical defense” in patients taking their medication as prescribed. Special concern exists for patients enrolled in methadone maintenance programs.

Population studies were conducted on representative samples of Spanish drivers in 2008/9, 2013 and 2015 [10, 11]. In the 2008/9 study [9], in the context of the European DRUID Project, the presence of drugs was determined in all drivers, whether they had tested positive in the roadside screening process or not. Nevertheless, in the 2013 and 2015 studies, confirmation analyses were only carried out for the cases that tested positive.

Within the DRUID project, to ensure that the results were comparable, analytical cut-offs were chosen that could be measured by all participating laboratories for each of the core substances [1]. Under the zero-tolerance approach, it is unlawful to drive with any amount of specified drugs in the body [3], even if it is too low and/or unlikely to produce any effects on driving skills. In the current study, the lower detection limit of a reference laboratory was used [10, 12], this being lower than the cut-offs used in the DRUID project [10, 11].

The goal of the study is two-fold: i) to determine the prevalence of opioids in Spanish drivers (heroin, morphine, codeine, methadone and tramadol); and ii) to explore if opioid drug usage is unaccompanied or in combination with other drugs, and if so, to what extent and at what concentrations.

## Methods

### Target population

Motor vehicle drivers on Spain’s public roads, excluding bikers and drivers of vehicles over 3500 kg.

### Design and database

Drivers were selected at random from the total population of drivers using a sampling scheme stratified by country areas, time period, population size, and road type, following the DRUID criteria as previously described [10, 11, 13]. A total of 128 police roadside checkpoints were selected. Roadside tests took place between September 26th, 2008, and August 24th, 2009. The data recorded in the Spanish DRUID database, which included information on a representative sample of Spanish drivers (*n* = 3302), has been re-analyzed.

### Procedure

Police officers carried out breath tests for alcohol using the Dräger Alcotest® 6810 device. For drugs, they used the on-site OF test, Dräger Drug Test® 5000. OF samples were screened and subsequently confirmed by LC-MS/MS quantification [10]. Consistent with the DRUID project [1, 2], various substances (drugs and some metabolites) were screened in each OF sample, including amphetamine, MDMA, MDA, MDEA, methamphetamine, cocaine, benzoylecgonine, delta 9 tetrahydrocannabinol (THC), THC-COOH, 6-acetylmorphine (6-AM), morphine, codeine, methadone, tramadol, hypnotics and sedatives (zolpidem, zopiclone, flunitrazepam), and anxiolytics (alprazolam, clonazepam, diazepam, lorazepam, nordiazepam, oxazepam). For this study, a positive OF result was defined as having a concentration higher than the lower limit of detection. Furthermore, breath alcohol was assessed in all cases, and a result was considered positive if ≥0.05 mg/L.

### Variables

- Sociodemographic data: gender, age, kilometers driven.

- The prevalence of opioids in drivers: If opioids were detected (6-acetylmorphine, morphine, codeine, methadone or tramadol), they were recorded as positive cases and later categorized as i) an opioid detected alone, or ii) opioids in conjunction with other opioids or other substances.

- The concentrations were calculated for these five opioids (ng/ml).

- The prevalence of opioid-positive cases was calculated after weighting for traffic intensity, as earlier described [10, 13]. However, the other figures were calculated for data without being weighted for traffic intensity.

### Statistical analysis

Frequencies with 95% confidence intervals (CIs), mean and SD for age and kilometers driven, and median with percentiles 25 and 75 (Q1-Q3) for concentrations are shown. Differences between groups were determined for the categorical variables using Pearson’s Chi-squared test and, for the continuous variables, through the Mann-Whitney U test. Statistical analyses were carried out with the Statistical Package for the Social Sciences (SPSS, v19). The level of significance was set at *p* ≤ 0.05.

## Results

### Prevalence of opioids in drivers

Opioids were detected (weighted data from traffic exposure) in 1.8% [95% IC, 1.4–2.3] (*n* = 58) of the cases (males 1.6%, females 2.4%, Chi-square = 2.09, *p* > 0.05). They were middle aged (Mean ± SD, 37.54 ± 11.64) and drove an average of (mean ± SD) 333.53 ± 351.36 Km/week.

### Patterns of opioid consumption

Twenty eight out of 64 drivers who tested positive for some opioid (43.8%) (data not weighted for traffic exposure) only tested positive for one substance (codeine *n* = 10, methadone *n* = 10, 6-AM *n* = 8). In the remaining 36 cases, opioids were found along with other substances: in 22 cases (34.3%), two or more different opioids were detected, and in 20 out of 22 cases cocaine was also detected. In 14 drivers (21.9%), one opioid was found combined with other substances, but of those cases, 11 included the detection of cocaine. No drivers were detected with morphine alone: all of them also have 6-AM, which implies heroin use. No drivers were also detected with tramadol.

Methadone was detected in 24 drivers. The detected concentration (median ng/ml [Q1-Q3]) (Table 1) was 1.71 ng/ml [0.10–15.30], while differences were not observed between cases detected alone (*n* = 10) or with other substances (*n* = 14)(U-Mann Whitney = 59.00; *p* > 0.05).

**Table 1:** Opioids in drivers: Concentrations in oral fluid, laboratory confirmation data (ng/ml)

	Opioid Alone	Opioid in combination	Total
	median [with percentiles 25 and 75 (Q1-Q3)]	median [with percentiles 25 and 75 (Q1-Q3)]	median [with percentiles 25 and 75 (Q1-Q3])
6-AM	*n* = 8	*n* = 26	*n* = 34
	1.56	8.35	5.71
	[0.32–4.40	[3.65–200.00]	[1.53–84.05]
Morphine		*n* = 20	*n* = 20
		37.40	37.40
		[2.84–200.00]	[2.84–200.00]
Codeine	*n* = 10	*n* = 16	*n* = 26
	67.0	17.00	40.55
	[4.50–104.21]	[1.70–186.06]	[2.10–120.77]
Methadone	*n* = 10	*n* = 14	*n* = 24
	1.65	2.70	1.71
	[0.10–5.42]	[0.10–71.87]	[0.10–15.30]

*6-AM* 6-acetylmorphine

*THC* delta 9 tetrahydrocannabinol

Codeine was detected in 26 drivers, with a concentration of 40.55 ng/ml [2.10–120.77]. In 10 cases codeine was alone, but in 16 cases codeine was detected along with other substances (U-Mann Whitney = 59.00; *p* > 0.05).

Heroin’s main metabolite 6-AM was detected in the OF of 34 drivers. Morphine was also detected in 20 cases. In 8 drivers, only 6-AM was detected. In the remaining 26 cases, 6-AM was detected with cocaine and, in some of those cases, additional substances as well. When 6-AM (6-AM + morphine) was detected alone, the OF concentration of 6-AM (1.56 ng/ml [0.32–4.40]) was lower than in the cases in which 6-AM (6-AM + morphine) was found in combination with other substances (8.35 ng/ml [3.65–200.00])(U-Mann Whitney = 44.00; *p* < 0.01). The mean morphine concentration was 37.40 ng/ml [2.84–200.00].

As presented in Table 2, cocaine was the most frequently detected substance in drivers who tested positive for opioids (100% in heroin users, 87.5% in codeine-positive cases, and 78.6% among methadone users). THC was the second most commonly detected substance, followed by benzodiazepines (including zolpidem and zopiclone), alcohol and amphetamines.

**Table 2:** Opioids in drivers: The presence of alcohol and other drugs in drivers, cases in which opioids were detected together with other substances

	6-AM	Codeine	Methadone
	(*n* = 26)	(*n* = 16)	(*n* = 14)
	%	%	%
Cocaine	100	87.5	78.6
Amphetamines	7.7	18.5	21.4
THC	34.6	31.2	35.7
Benzodiazepines, zolpidem and zopiclone	15.4	31.2	28.6
Alcohol	15.4	31.2	14.3

*6-AM* 6-acetylmorphine

*THC* delta 9 tetrahydrocannabinol

## Discussion

Driving with the presence of opioids is relatively infrequent, but polydrug use is common, and in most cases opioids were detected in low concentrations. Regarding methadone, only 40% of positive cases (most likely those on methadone maintenance programs) were using this substance alone, and in most cases, at a very low concentration.

The frequency of opioid-positive cases (1.8% [95% IC, 1.4–2.3]) was more than three times higher than when the analytical cut-off from the DRUID project was used (6-acetylmorphine 16 ng/ml, codeine 94, methadone 22, morphine 95, tramadol 480) [1, 2] (0.45 for medicinal opioids and 0.33 for illicit opioids, 0.5% [0.3–0.8] when any opioid was considered) [11]. In the European DRUID project, 0.07% tested positive for illicit opioids (range in the 13 European countries, 0.0%–0.3%) and 0.35% for medicinal opioids (range 0.0%– 0.8%) [1]. Important differences were found between regions: Southern Europe showed higher figures for illicit opioids, and Northern Europe showed higher figures for medicinal opioids [1].

More than half of drivers (56.2%) tested positive for opioids and other drugs, specifically cocaine. The presence of multiple substances in drivers has been associated with a “greatly increased risk” (relative risk in the range of 5–30) [1].

The DRUID categorization system established and defined standardized and harmonized criteria to categorize commonly used medications based on their influence on fitness to drive. According to its influence on fitness to drive, a medicine could be categorized as follows [14]:

• Category 0 (none or negligible influence on fitness to drive),

• Category I (minor influence on fitness to drive),

• Category II (moderate influence on fitness to drive),

• Category III (severe influence on fitness to drive).

The effects of opioids (medicines included in the Anatomical Therapeutic Chemical classification, N02A) on cognitive and psychomotor performance tasks related to driving, as well as the profile of the unwanted effects of these drugs, distinguish opioids categorized as DRUID II or III [14]. However, this categorization is a tool for improving the prescription and dispensation of medicines and for the provision of appropriate information to the patient. Doses, duration of use, use with other psychotropic agents and/or alcohol, the clinical status of the patient, and the effects of disease or diseases need to be considered in each patient.

The chronic use of opioids and their effect on driving performance has been a topic of debate, especially in the treatment of pain [15–17]. As reported in a systematic review, no clear and overall statement on driving performance can be generalized to all patients [16]. Furthermore, in a recent DRUID-derived study, large inter-individual variations were observed, and the authors concluded that in everyday practice, individual assessments should be done and patients should always be informed on potential driving impairments due to opioid use [17].

Furthermore, buprenorphine (buprenorphine/naloxone) and methadone are available in most developed countries for the treatment of opioid dependence (Anatomical Therapeutic Chemical classification, N07 BC drugs used in opioid dependence). Methadone and buprenorphine (buprenorphine/naloxone) impair driving performance and are categorized as DRUID II or III [14]. Again, doses, duration of treatment, the disorder/substance use disorder, the frequency of the comorbidity situation, and the frequency of use of psychotropic/alcohol/illicit substances, etc., need to be taken into account before making individual assessments.

In a review on the topic [18], driver impairment (cognitive and psychomotor function) was observed in patients on either a methadone maintenance program or a buprenorphine maintenance program compared to control groups. The authors noticed that the patients included in the buprenorphine maintenance program were less impaired [18]. Again, as previously addressed, the chronic use of opioids in other medical conditions [15–17] and the effects of long-term therapy with buprenorphine and/or methadone on driving performance are not yet clear [19], but it seems that patients in a buprenorphine maintenance program on stable dosing show less impairment than healthy controls [20]. However, an increased risk for motor vehicle accidents has been reported for patients involved in either buprenorphine or methadone treatment [21–25].

The data show that in 4 out of 10 cases, methadone was detected alone. That is, in most cases, other substances were found. In many countries, methadone maintenance programs are frequently implemented. From the perspective of health professionals, this should be taken into account, because many of their patients drive motor vehicles [26, 27]. Therefore, patients need to be properly informed about the increased risk of accidents and also the possibility of being detected by law enforcement on the road. Although a methadone-prescribing physician in Spain can provide a report on a person’s current treatment, if another substance that is not medically prescribed is detected, the driver will be fined [4]. Furthermore, as this study shows, it is likely that patients on methadone maintenance programs are also taking other illicit drugs and are undergoing treatment with other psychotropic medications [19, 20].

This could be applied to morphine and codeine, which are frequently used as medicinal drugs. The use and misuse of opioids has increased in the last few years in many developed countries [28].

Worldwide, there are different approaches to address driving under the influence of drugs. Frequently, in Spain, “any illegal/illicit drug and/or metabolite(s) of such drug present in the blood of a driver suspected of impairment should constitute per se evidence of drugged driving” [29]. A zero-tolerance approach is used due to the difficulties in determining drug impairment and its relationship to body fluid concentration. There is still much debate over this approach, especially regarding cannabis, which is the most common illicit drug used by drivers [30, 31].

Although knowledge of opioid-related driving risk is improving, it may not be enough [1, 2, 21–25] to allow for the establishment of common, worldwide per se legislation. For example, in the UK [5], limits for opioids (micrograms per liter of blood) have been established, specifically for methadone (500), 6-monoacetylmorphine (5), and morphine (80). Similarly, Norway [6] has introduced per se limits for some substances, representing drug concentrations in whole blood that are likely to be accompanied by a degree of impairment comparable to a BAC of 0.02%, and limits for graded sanctions, representing drug concentrations in whole blood that are likely to induce impairment comparable to BACs of 0.05% and 0.12%. For morphine, these limits were respectively 9, 24 and 61 ng/ml in whole blood, but a limit of 25 ng/ml for methadone was established as impairment comparable to a BAC of 0.02%.

In our study, all cases that demonstrated methadone detection (a median concentration in OF of 1.71 ng/ml] showed very low concentrations of the drug. If we apply the cut-offs used in Norway (a limit of 25 ng/ml in whole blood was established as impairment comparable to a BAC of 0.02%), only very few positive cases would have been established. If we apply the cut-offs from the United Kingdom (500 micrograms per liter of blood), no positive case would have been found (in both cases, using the DRUID project equivalent with analytical cut-off values for methadone of 10 (ng/ml) in whole blood and 22 (ng/ml) in oral fluid, although this comparison should be interpreted tentatively because the correlation between blood and OF is not well established [1, 2]).

With the current zero-tolerance approach in Spain, a significant proportion of the population screened at random for driving under the influence of drugs could be fined (1.8%), generally with low concentrations. In our opinion, a per se law with pre-established cut-off values would be more reasonable, but avoiding alcohol and drug-driving should be our goal and the implementation of appropriate measures is a priority worldwide [3]. Reaching an international perspective on the applied legislation is not easy [3, 32]. Norway’s approach, in which graded sanctions are established (when possible) based on equivalency or comparability to various BACs [6], seems more rational for us. In our opinion, although zero-tolerance laws are easily understood by the population, this approach could be difficult to maintain long-term as driving with a certain amount of alcohol in our body is allowed. Our study shows that 6 out of 10 drivers with methadone in their OF (those most likely on methadone maintenance programs) are using other substances. This should be considered by prescribing physicians and other health professionals in order to properly inform patients about the added risk on the road when mixing substances.

The database used here is unique because the presence of drugs was determined in all drivers, whether or not they had tested positive in the roadside screening process, and because it included a representative sample of the Spanish driver population [10, 13]. However, the extent to which these figures and conclusions could be applied to other countries should be viewed with caution. Finally, the data are from 2008/9, which implies that changes over time could not be discharged. Nevertheless, a comparison between the 2008/9 study and the 2013 study shows no statistical difference in the opioid prevalence between these years [11].

## Conclusions

This study shows that driving with the presence of opioids in the body is relatively infrequent among Spanish drivers. When opioids are present, they are frequently detected with several substances at the same time and mostly at low concentrations. These findings also apply to the methadone-positive cases: 6 out of 10 drivers with methadone in their OF were using other substances. Opioids can be used illegally or medically prescribed. Health professionals should consider that opioids effect tasks related to driving and are associated with increased road traffic accidents, most likely when mixed with other substances. Health professionals, particularly those involved in the treatment and follow-up of patients with opioid use disorders, should inform their patients about the possibility of these substances being detected in roadside tests and the increased risks that they face when driving with opioids in their body.

## Abbreviations

6-AM: 6-acetylmorphine
OF: Oral Fluid
THC: delta 9 tetrahydrocannabinol
UK: United Kingdom

## References

[1] Schulze H, Schumacher M, Urmeew R, Auerbach K, Alvarez FJ, Bernhoft IM, de Gier H, Hagenzieker M, Houwing S, Knoche A, Pilgerstorfer M, Zlender B (2012). Final report: work performed, main results and recommendations. Driving under the influence of drugs, alcohol and medicines in Europe — findings from the DRUID project. Publications Office of the European Union.

[2] DRUID (2012). Final report: work performed, main results and recommendations. Revisión 2.0.

[3] WHO (2016). Drug use and road safety: a policy brief.

[4] Álvarez FJ, González-Luque JC, Seguí-Gómez M (2015). Drugs, substance use disorder and driving: intervention of health professionals in the treatment of addictions. Adicciones.

[5] GOV:UK. Drugs and driving: the law. Collection Drug driving. https://www.gov.uk/drug-driving-lawhttps://www.gov.uk/government/collections/drug-driving#table-of-drugs-and-limits. Accessed 9 Jan 2017.

[6] Norwegian Ministry of Transport and Communications (2014). Driving under the influence of non-alcohol drugs – legal limits implemented in Norway.

[7] Verstraete AG. Oral fluid testing for driving under the influence of drugs: history, recent progress and remaining challenges. Forensic Sci Int 2005;150:143-50. 10.1016/j.forsciint.2004.11.023 15944054

[8] Gjerde H, Langel K, Favretto D, Verstraete AG (2014). Estimation of equivalent cutoff thresholds in blood and oral fluid for drug prevalence studies. J Anal Toxicol.

[9] Department for Transport (2014). Guidance for healthcare professionals on drug driving.

[10] Gómez-Talegón T, Fierro I, González-Luque JC, Colás M, López-Rivadulla M, Álvarez FJ (2012). Prevalence of psychoactive substances, alcohol, illicit drugs, and medicines, in Spanish drivers: a roadside study. Forensic Sci Int.

[11] Fierro I, González-Luque JC, Seguí-Gómez M, Álvarez FJ (2015). 2015. Alcohol and drug use by Spanish drivers: comparison of two cross-sectional road-side surveys (2008-9/2013). Int J Drug Policy.

[12] Concheiro M, de Castro A, Quintela O, Cruz M, López-Rivadulla M (2008). Determination of illicit and medical drugs and their metabolites in oral fluid and preserved oral fluid by liquid chromatography-tandem mass spectrometry. Anal Bioanal Chem.

[13] Fierro I, González-Luque JC, Alvarez FJ (2014). The relationship between observed signs of impairment and THC concentration in oral fluid. Drug Alcohol Depend.

[14] Ravera S, Monteiro S, de Gier JJ, van der Linden T, Gómez-Talegón T, Álvarez F (2012). J. & the DRUID project WP4 partner. A European approach to categorising medicines for fitness to drive: outcomes of the DRUID project. Br J Clin Pharmacol.

[15] Kendall SE, Sjøgren P, de Pimenta CAM, Højsted J, Kurita GP (2010). The cognitive effects of opioids in chronic non-cancer pain. Pain.

[16] Mailis-Gagnon A, Lakha SF, Furlan A, Nicholson K, Yegneswaran B, Sabatowski R (2012). Systematic review of the quality and generalizability of studies on the effects of opioids on driving and cognitive/psychomotor performance. Clin J Pain.

[17] Schumacher MB, Jongen S, Knoche A, Petzke F, Vuurman EF, Vollrath M, Ramaekers JG (2017). Effect of chronic opioid therapy on actual driving performance in non-cancer pain patients. Psychopharmacology.

[18] Rapeli P, Fabritius C, Kalska H, Alho H (2011). Cognitive functioning in opioid-dependent patients treated with buprenorphine, methadone, and other psychoactive medications: stability and correlates. BMC Clin Pharmacol.

[19] Strand MC, Fjeld B, Arnestad M, Mørland J (2013). Can patients receiving opioid maintenance therapy safely drive? A systematic review of epidemiological and experimental studies on driving ability with a focus on concomitant methadone or buprenorphine administration. Traffic Inj Prev.

[20] Shmygalev S, Damm M, Weckbecker K, Berghaus G, Petzke F, Sabatowski R (2011). The impact of long-term maintenance treatment with buprenorphine on complex psychomotor and cognitive function. Drug Alcohol Depend.

[21] Corsenac P, Lagarde E, Gadegbeku B, Delorme B, Tricotel A, Castot A, Moore N, Philip P, Laumon B, Orriols L (2012). Road traffic crashes and prescribed methadone and buprenorphine: a French registry-based case-control study. Drug Alcohol Depend.

[22] Bramness JG, Skurtveit S, Mørland J, Engeland A (2012). An increased risk of motor vehicle accidents after prescription of methadone. Addiction.

[23] Elvik R (2013). 2013. Risk of road accident associated with the use of drugs: a systematic review and meta-analysis of evidence from epidemiological studies. Accid Anal Prev..

[24] Kuypers KP, Legrand SA, Ramaekers JG, Verstraete AG. 2012. A case-control study estimating accident risk for alcohol, medicines and illegal drugs. PLoS ONE. 2012;7:e43496. 10.1371/journal.pone.0043496 PMC3429508 22952694

[25] Rudisill TM, Zhu M, Kelley GA, Pilkerton C, Rudisill BR (2016). Medication use and the risk of motor vehicle collisions among licensed drivers: a systematic review. Accid Anal Prev.

[26] Roncero C, Álvarez FJ, Barral C, Gómez-Baeza S, Gonzalvo B, Rodríguez-Cintas L, Brugal MT, Jacas C, Romaguera A, Casas M (2013). PROTEUS study investigators. Driving and legal status of Spanish opioid-dependent patients. Subst Abuse Treat Prev Policy.

[27] Soyka M (2014). Opioids and traffic safety – focus on Buprenorphine. Pharmacopsychiatry.

[28] NIDA. Topics in brief. Prescription Drug Abuse – December 2011. https://www.drugabuse.gov/sites/default/files/prescription_1.pdf. Accessed 9 Jan 2017.

[29] Reisfield GM, Goldberger BA, Gold MS, DuPont RL (2012). The mirage of impairing drug concentration thresholds: a rationale for zero tolerance per se driving under the influence of drugs laws. J Anal Toxicol.

[30] Wong K, Brady JE, Li G (2014). Establishing legal limits for driving under the influence of marijuana. Inj Epidemiol.

[31] Wolff K, Johnston A (2014). Cannabis use: a perspective in relation to the proposed UK drug-driving legislation. Drug Test Anal.

[32] EMCDDA. Legal approaches to drugs and driving. http://www.emcdda.europa.eu/html.cfm/index19034EN.html. Accessed 9 Jan 2017.

